# Modeling the potential risk factors of bovine viral diarrhea prevalence in Egypt using univariable and multivariable logistic regression analyses

**DOI:** 10.14202/vetworld.2018.259-267

**Published:** 2018-03-02

**Authors:** Abdelfattah M. Selim, Mahmoud M. Elhaig, Sherif A. Moawed, Ehab El-Nahas

**Affiliations:** 1 Department of Animal Medicine (Infectious Diseases), Faculty of Veterinary Medicine, Benha University, P.O. Box 13736, Toukh, Egypt; 2 Department of Animal Medicine (Infectious Diseases), Faculty of Veterinary Medicine, Suez Canal University, Ismailia 41522, Egypt; 3 Department of Animal Wealth Development, Biostatistics Division, Faculty of Veterinary Medicine, Suez Canal University, Ismailia 41522, Egypt; 4 Department of Virology, Faculty of Veterinary Medicine, Benha University, P.O. Box 13736, Toukh, Egypt

**Keywords:** bovine viral diarrhea, likelihood ratio test, logistic regression, odds ratio, seroprevalence

## Abstract

**Aim:**

The present cross-sectional study was conducted to determine the seroprevalence and potential risk factors associated with Bovine viral diarrhea virus (BVDV) disease in cattle and buffaloes in Egypt, to model the potential risk factors associated with the disease using logistic regression (LR) models, and to fit the best predictive model for the current data.

**Materials and Methods:**

A total of 740 blood samples were collected within November 2012-March 2013 from animals aged between 6 months and 3 years. The potential risk factors studied were species, age, sex, and herd location. All serum samples were examined with indirect ELIZA test for antibody detection. Data were analyzed with different statistical approaches such as Chi-square test, odds ratios (OR), univariable, and multivariable LR models.

**Results:**

Results revealed a non-significant association between being seropositive with BVDV and all risk factors, except for species of animal. Seroprevalence percentages were 40% and 23% for cattle and buffaloes, respectively. OR for all categories were close to one with the highest OR for cattle relative to buffaloes, which was 2.237. Likelihood ratio tests showed a significant drop of the −2LL from univariable LR to multivariable LR models.

**Conclusion:**

There was an evidence of high seroprevalence of BVDV among cattle as compared with buffaloes with the possibility of infection in different age groups of animals. In addition, multivariable LR model was proved to provide more information for association and prediction purposes relative to univariable LR models and Chi-square tests if we have more than one predictor.

## Introduction

Bovine viral diarrhea virus (BVDV), the causal agent of BVD and mucosal disease complex, is classified in the genus Pestivirus in the family Flaviviridae. Although cattle are the primary host for BVDV, several reports suggest most even-toed ungulates are also susceptible. It causes important economic losses in cattle breeding. Infection is characterized by depression, temperature, mild diarrhea, and temporary leukopenia [1].

Serologic surveys indicate that BVDV is distributed worldwide. The prevalence of antiviral antibody in cattle varies among countries and may vary between geographic regions within a country. Prevalence of antiviral antibody may be >90% if vaccination is practiced commonly in a geographic region. Although cattle of all ages are susceptible, most cases of the overt clinical disease are seen in cattle between 6 months and 2 years old [2].

Cattle that are persistently infected (PI) with noncytopathic BVDV serve as a natural reservoir for virus. Persistent infection develops when noncytopathic BVDV is transmitted transplacentally during the first 4 months of fetal development. The calf is born infected with virus, remains infected for life, and usually is immunotolerant to the resident noncytopathic virus [3]. Transplacental infection that occurs later in gestation results in abortion, congenital malformations, or birth of normal calves that have antibody against BVDV. The prevalence of persistent infection varies among countries and between regions within a country [4].

PI animals result from the infection of the bovine fetus with an NCP-BVDV biotype early in gestation. These animals show specific immunological tolerance to the carrier virus and maybe born apparently healthy. PI animals are the main source of virus transmission as they continuously shed large amounts of virus in the environment. Virus is excreted in smaller amounts from acutely infected animals and for only a few days during the acute infection [5].

Early detection of antibodies using enzyme-linked immunosorbent assay (ELISA) is unreliable and difficult attached with appropriate antigen [6]. However, this has been overcome, resulting in Ab ELISAs with high specificity and sensitivity of up to 99% and 98%, respectively, when compared with the serum neutralization test (SNT) [7,8]. ELISA can detect various types of samples and are an efficient and economical alternative to SNT [9]. SNT is more sensitive than ELISA and can detect more antibodies following vaccination [10]. Furthermore, low SNT titer appeared in prolonged storage or repeated freeze-thawing samples or sample was negative with ELISA [11]. The objectives of this study were to determine the prevalence of BVDV in cattle and buffaloes in some localities in Egypt, to model the potential risk factors associated with BVDV prevalence using logistic regression (LR), and to fit the best predictive model for the current data.

## Materials and Methods

### Ethical approval

This study was conducted according to ethical guidelines approved by ethics of scientific research committee, Faculty of Veterinary Medicine, Suez Canal University, Ismailia, Egypt.

### Animal and sampling

A total of 480 and 260 blood samples were collected from cattle and buffaloes of from four governorates (Kalubia, Giza, Menofia, and Gharbia) in Egypt. Samples for examination of BVDV antibodies were collected from animals aged between 6 months and 3 years. The samples were collected from apparently healthy and diseased animals without a history of vaccination. All blood samples were collected within the period November 2012-March 2013. The age, sex, species, and location of the animal were studied for being potential risk factors for BVD seropositivity.

### Indirect ELISA

The all collected serum samples were examined with indirect ELISA test kit (Svanova BVDV antibody ELISA, Svanova Biotech AB). The tests were performed following the manufacturer’s instructions. The antibody titer was interpreted on the basis of the percentage positivity by dividing the sample OD values by positive reference sample OD values. The cutoff value was set at 14%.

### Statistical analyses and models

#### Nonparametric analysis

Data were analyzed statistically to test the potential association between the BVDV occurrence and each of the predictors (gender, age, species, and herd location) using nonparametric Chi-square tests. Although Chi-square test allows testing these relationships, the nonparametric test has some limitations; first, Chi-square test did not permit for the potential effect of other independent variables on that relationship. Second, Chi-square test was not able to provide a predictive model for future prediction of the outcome. Third, Chi-square test did not assess the relationship between a dependent categorical variable and several predictors at the same time. Fourth, the magnitude and contribution of each predictor in explaining the outcome cannot be calculated by Chi-square test. Therefore, it is necessary to search for another approach, the binary LR analysis being used as a potential alternative statistical test for analysis of categorical outcomes.

### LR analysis

#### Fitting the LR models

Data were also analyzed using univariable and multivariable LR techniques for modeling the potential risk factors related to BVD disease and, to explain more practical and statistical facts that could not be denoted by the ordinary Chi-square test. The univariable LR models were fitted using only one explanatory variable along with examining its relationship with the outcome of BVD disease. The multivariable LR model was applied to assess the relationship between a dichotomous outcome and many explanatory variables. The contribution of each predictor variable in explaining the outcome was measured by the LR coefficients and odds ratio (OR). In univariable LR, the natural log odds of BVDV was fitted as a linear function of the predictors [12] as follows:


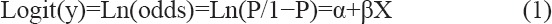


Where p is the probability of outcome variable; X is the predictor variable; α and β are the LR coefficients. An equation for predicting the probability of BVD occurrence was obtained by shifting Equation (1) using the antilog on both sides as follows:

P=Probability (y=BVD outcome/X=x, a specified value)


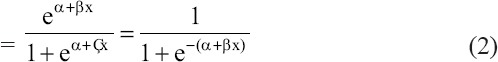


Where p is the probability of the BVD outcome, α is the Y-intercept, β is the regression coefficient, and e is the base of the natural logarithm (e=2.71828). The relationship between logit (Y) and X in Equation (1) is linear, while the relationship between the probability of Y and X in Equation (2) is nonlinear. Hence, the natural log transformation of the odds of BVD was imperative to exhibit the linear relationship between the dependent and independent variables. By the way, the multivariable LR model was fitted as follows:


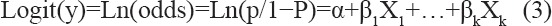


Therefore,


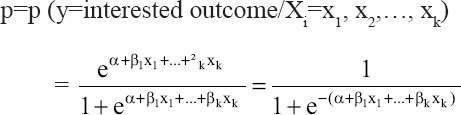


### Evaluation of LR models

#### Overall model evaluation

Likelihood ratio test (LRT) was used to compare the null model with tested models, where one or more predictor variables were incorporated in the model. LRT test the null hypothesis:


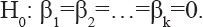


The deviation with the null model (−2 log likelihood [−2LL] of null model) was compared to the corresponding term for a given model with explanatory variables (−2 log L of the given model). The differences between the two −2 log L produce the Chi-square statistic (χ^2^), with k degrees of freedom [13].

The probability level < 0.05 suggests that the given model work well and better than the null model. Hence, at least one of the predictor variables participates in explaining and predicting the outcome.

Hosmer-Lemeshow test was applied for assessing the goodness of fit of LR models. The test depends mainly on the same principle of Chi-square test of testing the differences between observed and predicted frequencies. The value of test statistics [14] was calculated as:


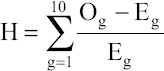


Where O_g_ and E_g_ are the observed and expected values for the gth deciles. Hosmer-Lemeshow test follows a χ^2^ distribution with 8 degrees of freedom (df) (number of groups-2). p>0.05 suggests a good fit of data by the LR model.

#### Testing the significance of LR coefficients

Wald statistic was used to test the significant contribution of each predictor in the given LR model. The Wald statistic also distributed with Chi-square and calculated as the ratio of the square of regression slope to the square of the standard error of that coefficient [13]:


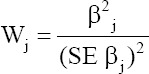


For the present data, the Wald statistic was tested at degrees of freedom equal to the number of categories of the X variable −1. The predictor variable was considered significantly affect the outcome of BVD if p≤0.05. All statistical analyses were performed by Statistical Package for the Social Sciences (SPSS version 20) and Statistical Analysis System (SAS Institute) software. Results are considered significant at a probability level of 0.05 for each p≤0.05.

## Results and Discussion

### Association between BVD prevalence and potential risk factors using Chi-square test

Table-1 showed the association between the condition of BVDV and gender. Chi-square test (0.480) revealed insignificant (p>0.05) association between BVDV infection and gender. Odds of infection were found to be 0.553 for males and 0.495 for females. That is, the OR for male relative to female was 1.117, indicating the absence of association (OR was close to one). Table-2 gave the relationship between age and the seroprevalence of BVDV. The age group <6 months was incorporated as a baseline category because it had a code of zero in the dataset. The infection with BVDV was non-significantly (p>0.05) associated with the age (Chi-square=0.239). The results showed similar odds of BVDV infection with increasing age of the animal. The OR of the two age groups other than the baseline were nearly equal (close to one), suggesting that all age groups would be a potential risk factor for BVDV disease. A similar study was conducted by Talafha *et al*. [15] who reported non-significant differences in BVDV seroprevalence between different age groups.

**Table-1:** Chi-square test for the association between BVD prevalence and gender.

Gender	Coded values in dataset	BVDV condition (%)		Total	Odds of BVDV infection

		Positive (1)	Negative (0)		
Male	1	99 (35.6)	179 (64.4)	278	99/179=0.553
Female	0	152 (33.1)	307 (66.9)	459	152/307=0.495
Total		251	486	737	Odds ratio=0.553/0.495=1.117

Chi-square test was done through the cross-tabulation (gender * BVDV disease) option of SPSS using the original raw data of the individual cases. The test result denoted the value of Chi-square as 0.480 at df=1 along with the probability value of 0.488 (>0.05). BVDV=Bovine viral diarrhea virus, SPSS=Statistical Package for the Social Sciences, df=degrees of freedom

**Table-2:** Chi-square test for the association between BVD prevalence and age.

Age groups (months)	Coded values in dataset	BVDV condition (%)		Total	Odds of BVDV infection	Odds ratio compared to baseline age

		Positive (1)	Negative (0)			
<6 months	0 (baseline)	45 (35.2)	83 (64.8)	128	45/83=0.542	1.0
6-12 months	1	86 (33.0)	175 (67.0)	261	86/175=0.491	0.491/0.542=0.906
>12 months	2	120 (34.5)	228 (65.5)	348	120/228=0.526	0.526/0.542=0.970
Total		251	486	737		

Chi-square test was done through the cross-tabulation (age * BVDV disease) option of SPSS using the original raw data of the individual cases. The test result denoted the value of Chi-square as 0.239 at df=2 along with the probability value of 0.887 (>0.05). BVDV=Bovine viral diarrhea virus, SPSS=Statistical Package for the Social Sciences, df=degrees of freedom

When the sex was determined or provided, approximately two-thirds of the specimens were from females. This likely reflects that bovine females outnumber males, and especially in the case of dairy cattle, are often of greater financial value. The non-significant difference in prevalence of BVD in relation to sex of examined animals in this study come in accordance with other previous studies as Wilson *et al*. [16], where the % of infection was 2.3% in male and 2.4% in female. In contrast, some studies reported a significant difference in prevalence of BVD between male and female as Bello *et al*. [17], the prevalence rate was 75% in female and 59.3% in male.

The occurrence of BVD in the present study according to age group was more prevalent in animals <6 and those over than 12 months. This finding is in agreement with Wilson *et al*. [16], they reported prevalence rate in calf, juvenile, and adult as 5%, 2.2%, and 5.3%, respectively. Out of a total of 157 cows within 18 dairy cattle herds in a suburb of Mashad, Iran, 57 (36.3%) were calves, 36 (22.9%) were heifers, and 64 (40.8%) were adult dairy cows [18]. Furthermore, Kish *et al*. [19] and Sayers *et al*. [20] found the prevalence rate more in juvenile animals than adults’ animals. The prevalence rate increases in young animals at risk [21,22].

In contrast, the prevalence rate of BVD in animals of age above 3 years (79%) is higher than young animals with age <1 year (70%) as previously reported by Vásquez *et al*. [23]. The lower prevalence rate in calve may be due to some calves might be persistent infected animal and immunotolerant to BVD virus which not produce antibodies and cannot be detected by serological tests [24-26].

The prevalence of BVD in the two studied species was observed to be higher in cattle (40%) than buffaloes (23.0%). The odd of infection for buffaloes was 0.298 compared to 0.667 for cattle. This implies that the OR for cattle versus buffaloes was 2.237 (1/0.447) and subsequently, it can be concluded that the odds of BVDV for cattle were 2.237 times the odds of BVDV for buffaloes. This result was confirmed by the value of Chi-square (21.648) which indicates a highly significant (p≤0.01) association between prevalence of BVD and species (Table-3). Because cattle are the most reared breed in Egypt in comparison with buffaloes, due to its high milk yield, it showed a higher prevalence of BVD. This result agrees with other previous studies [17,19,27].

**Table-3:** Chi-square test for the association between BVD prevalence and species.

Species	Coded values in dataset	BVDV condition (%)		Total	Odds of BVDV infection

		Positive (1)	Negative (0)		
Buffalo	1	59 (23.0)	198 (77.0)	257	59/198=0.298
Cattle	0	192 (40.0)	288 (60.0)	480	192/288=0.667
Total		251	486	737	Odds ratio=0.298/0.667=0.447

Chi-square test was done through the cross-tabulation (species * BVDV disease) option of SPSS using the original raw data of the individual cases. The test result denoted the value of Chi-square as 21.648 at df=1 along with the probability value of 0.001 (<0.01). BVDV=Bovine viral diarrhea virus, SPSS=Statistical Package for the Social Sciences, df=degrees of freedom

The herd location had a non-significant (p>0.05) effect of on the BVD prevalence in Egypt, according to the Chi-square test result (Table-4). This result is in accordance with the finding of Talafha *et al*. [15]. The highest % of BVD prevalence was recorded for Kalubia province (38.9%). The odds of infection showed small differences among all localities. For this reason, Kalubia was selected as a baseline category and coded as zero in the dataset. The ORs were all less than one, and estimated to be; 0.675, 0.786, and 0.792, for Giza, Menofia, and Gharbia provinces, respectively, compared to Kalubia. In other words, the odds of BVD prevalence for Giza, Menofia, and Gharbia were 1.48 (1/0.675), 1.27 (1/0.786), and 1.26 (1/0.792), respectively, times less than the odds of BVDV occurrence for Kalubia. The present findings agree with Ghazi *et al*. [27] and El-Bagoury *et al*. [28]. In areas that had very high BVD seroprevalence, like those observed in this study, and where cattle density was high and herds clearing the infection were obviously at higher risk of reinfection from losing immune protection and becoming naive to the virus. Therefore, the vaccination of susceptible herds in combination with removal of PI animals, would overcome the problem of reinfection by preventing intrauterine infection in pregnant dams at risk of exposure to contact with undetected PI animals [29,30].

**Table-4:** Chi-square test for the association between BVD prevalence and localities.

Area (location)	Coded values in dataset	BVDV condition (%)		Total	Odds of BVDV infection	Odds ratio compared to baseline age

		Positive	Negative			
Kalubia	0 (baseline)	70 (38.9)	110 (61.1)	180	70/110=0.636	1.0
Giza	1	45 (30.0)	105 (70.0)	150	45/105=0.429	0.429/0.636=0.675
Menofia	2	71 (33.3)	142 (66.7)	213	71/142=0.50	0.50/0.636=0.786
Gharbia	3	65 (33.5)	129 (66.5)	194	65/129=0.504	0.504/0.636=0.792
Total		251	486	737		

Chi-square test was done through the cross-tabulation (farm location * BVDV disease) option of SPSS using the original raw data of the individual cases. The test result denoted the value of Chi-square as 3.047 at df=3 along with the probability value of 0.385 (>0.05). BVDV=Bovine viral diarrhea virus, SPSS=Statistical Package for the Social Sciences, df=degrees of freedom

### LR analysis

In the previous results, Chi-square test did not study the influence of other independent variables on that relationship. Hence, univariable and multivariable LR models were fitted to model and predict the potential risk factors with BVDV disease. The univariable LR models were created for a single predictor, followed by a multivariable LR model, controlling for the other independent variables. Moreover, LR models were imperative to quantify the coefficient estimates (the change in log Y for a one unit change in X), and to determine the direction of relationship between the predictor and the logit of outcome. The summary of univariable (Model 1, 2, 3, and 4) and multivariable (Model 5) LR models were presented in Table-5. The five models were:

**Table-5:** Univariable and multivariable Logistic regression models for modeling and predicting BVDV seroprevalence.

Model	Parameter	β	SE (β)	Wald statistic	df	p value	Exp^β^ (OR)	95% CI (OR)	

								Lower	Upper
Null model	Constant	−0.661	0.078	72.264	1	0.001	0.516		
Model 1	Gender	0.111	0.160	0.480	1	0.488	1.117	0.817	1.528
	Constant	−0.703	0.099	50.239	1	0.001	0.495		
Model 2	Age			0.239	2	0.887			
	Age (1)	−0.098	0.227	0.187	1	0.665	0.906	0.581	1.415
	Age (2)	−0.030	0.217	0.019	1	0.891	0.971	0.635	1.485
	Constant	−0.612	0.185	10.935	1	0.001	0.542		
Model 3	Species	−0.805	0.175	21.136	1	0.001	0.447	0.317	0.630
	Constant	−0.405	0.093	18.939	1	0.001	0.667		
Model 4	Area			3.035	3	0.386			
	Area (1)	−0.395	0.235	2.835	1	0.092	0.673	0.425	1.067
	Area (2)	−0.241	0.211	1.307	1	0.253	0.786	0.520	1.188
	Area (3)	−0.233	0.216	1.172	1	0.279	0.792	0.519	1.208
	Constant	−0.452	0.153	8.739	1	0.003	0.636		
Model 5	Gender	0.090	0.164	0.303	1	0.582	1.095	0.793	1.510
	Age			0.212	2	0.899			
	Age 1	−0.094	0.232	0.166	1	0.684	0.910	0.578	1.433
	Age 2	−0.029	0.221	0.017	1	0.897	0.972	0.630	1.499
	Species	−0.818	0.177	21.397	1	0.001	0.441	0.312	0.624
	Area			3.153	3	0.369			
	Area 1	−0.398	0.239	2.773	1	0.096	0.671	0.420	1.073
	Area 2	−0.277	0.215	1.665	1	0.197	0.758	0.498	1.155
	Area 3	−0.165	0.221	0.559	1	0.455	0.848	0.550	1.307
	Constant	−0.187	0.245	0.586	1	0.444	0.829		

Where: β is the logistic regression coefficient for independent variable, SE (β) is the standard error of coefficient, df is the degree of freedom, Exp^β^ (OR) is the estimated odds ratio, and−2 Log L is the Log-likelihood statistic. For better modeling and fitting of data, a number of P+1 model were fitted, where; P (= 4) is the number of predictor variables. CI is confidence interval

Log odds of BVD=−0.661 (The null model)

Log odds of BVD=−0.703+0.111*gender (Model 1)

Log odds of BVD= −0.612−0.098*age (1)−0.030* age (2) (Model 2)

Log odds of BVD=−0.405−0.805*species (Model 3)

Log odds of BVD= −0.452−0.395*area (1) −0.241*area (2)−0.233*area (3) (Model 4)

Log odds of BVD= −0.187+0.090*gender−0.094*age (1)−0.029*age (2)−0.818*species −0.398*area (1)−0.277*area (2) −0.165*area (3) (Model 5)

### Testing the significance of predictors

According to Model 1, the Wald Chi-square statistic revealed that gender was non-significant (p>0.05) predictor for the BVD prevalence. The intercept was significant (p<0.01), suggesting its important inclusion in the model. The log odds that an animal of a given gender would show the infection with BVD can be predicted from Model 1. For a male animal (gender=1), the log odds of being infected with BVD were −0.592. In practice, the value of log odds (−0.592) could be transformed using the base of exponential function [31]. Hence, the odds of BVD prevalence for male were 0.553 (=EXP−0.592). By the way, the log odds of a female animal (gender=0) are −0.703. Then, the odds of BVD occurrence for a female animal were 0.495 (=EXP−0.703). The OR was equal to 1.117 (odds of male/odds of female)=(0.553/0.495). Due to the previous complicated algebraic calculations, the value of OR (1.117) was predicted by the model (Table-5) through the base of exponent function (e=2.718) raised to the model coefficients (e^β^). The OR for gender was positive and close to one, suggesting that the coefficient of gender should be positive (0.111). The prevalence of BVD for each gender can be predicted in the form of percentages. For males, the odds of infection converted into probabilities as odds/1+odds to be 0.356 (=0.553/1.553). That is, Model 1 predicted that 35.6 % of males showed the seropositivity with BVDV. By the same way, the predicted probability for females was equal to 0.331 (=0.495/1.495). Model 1 predicted that 33.1% of female animals showed infection with BVD. In conclusion, the percent of males and females being infected with BVD predicted by the Model 1 were equal to those calculated in Table-1.

The univariable logit model (Model 2) showed age (1), the dummy variable for the second category of age (6-12 months), and age (2), the dummy variable for the third category of age (> 12 months), that would be compared with the age group <6 months. The Wald statistic revealed that age was not significant (p>0.05) predictor for the BVD occurrence (Table-5). Specifically, the Wald statistics for the age group 6-12 months and age group >12 months were 0.187 and 0.019, suggesting the absence of significant difference (p>0.05) between each of these two age groups relative to the baseline group (<6 months). The Wald statistic for the intercept testing was 10.935, which was also significant (p<0.01), reflecting the important contribution of intercept term in Model 2. By the same way of calculations in Model 1, the predicted probabilities for the three age groups of being infected with BVD were 35.2%, 33%, and 34.5%, respectively. It was obvious that the percentages estimated by the univariable logit Model 2 were similar to those in association table of Chi-square analysis (Table-2). Moreover, the OR for age group 6-12 months relative to the baseline group was 0.906 (=0.492/0.542), while the OR for age group >12 months relative to the reference group was 0.970 (=0.526/0.542). The two estimated ORs were the same as calculated in Table-2. Although all Wald statistics concerned with testing the significant effect of age were non-significant (p>0.05), the estimated ORs were <1, indicating a negative relationship between age categories and odds of BVD prevalence. This conclusion has been confirmed by the negative signs of coefficients in LR Model 2.

The univariable LR model (Model 3) associated with species showed a highly significant (p<0.01) relationship between species and outcome of BVDV disease. The model predicted that 23% (=0.298/1.298) of buffaloes showed the seropositivity of BVD (as in Table-3). Furthermore, Model 3 predicted that 40% (=0.667/1.667) of dairy cattle were positively infected with BVD. The OR of infection for buffaloes relative to cattle was 0.447 (0.298/0.667). The reciprocal of that value (1/0.447) resulted in the OR of cattle relative to buffaloes.

In Model 4, all the model coefficients were negative, suggesting negative associations with the studied outcome (Table-5). Regarding the estimated model, area (1) was the dummy variable for Giza, area (2) was the dummy variable for Menofia, and area (3) was the dummy variable for Gharbia. All these localities were compared with the reference area, Kalubia. The Wald statistic (3.035) for herd location was also non-significant (p>0.05), concluding no association between the seroprevalence of BVDV and localities. In addition, the Wald statistics for all dummy variables were non-significant (p>0.05). Results showed the significance (p<0.01) of intercept in Model 4. Using the formula (odds/odds+1), the probabilities predicted by Model 4 of seropositive infection with BVD were 38.9%, 30%, 33.3%, and 33.5%, respectively. The ORs for Giza, Menofia, and Gharbia localities relative to Kalubia were 0.673, 0.786, and 0.792, respectively.

The previous results were aimed to study the association and prediction of BVD outcome from a single predictor only. Before constructing the multivariable LR model (Model 5), multicollinearity among the independent variables was checked using multicollinearity diagnostic tests. The values of variance inflation factor (VIF=1/tolerance) were all <10, indicating the absence of collinearity between explanatory variables. It is imperative to mention that if VIF >5 or 10, the estimated coefficients will be invalid because of multicollinearity, which in turn lead to inflation of variances [32], and consequently inaccurate estimates and unreal inferences about the relationship between the explanatory variables and outcome [33]. Moreover, the absolute correlation coefficients were low (0.006-0.083) and the condition indices were <15.

Unlike univariable logit models, the intercept in the full model (Model 5) was non-significant (p>0.05), may be due to the inclusion of all predictors with dummies in the model. Furthermore, the negative values of regression coefficients indicate that the odds and the probability of BVD disease may decrease because both values of regression coefficient and OR are correlated and dependent. The estimated confidence intervals (95% CIs) for all ORs included one in its range, for all studied factors, except for species, suggesting that the association between BVD positivity and species was statistically significant at 0.05, because, the 95% CIs for species were 0.317-0.630 and 0.312-0.624 for Model 3 and Model 5, respectively. A 95% CI including the value one indicates the absence of significant association as reported by Szumilas [34]. According to the estimates of Model 5, it was observed that multivariable logit model resulted in very little effects on ORs and their interpretations. Furthermore, the direction of relationship between BVD outcome and predictors was the same. In a conclusion, the multivariable LR model yielded stable estimates, together with the absence of multicollinearity.

### Overall evaluation and goodness of fit of models

#### Predicted model relative to null model

LR models depend on the maximum likelihood (ML) estimators to assess the relationship between the outcome and predictor(s). The ML estimator relies on its ability to provide a model with a high degree of precision, for predicting an outcome [35]. A better LR model is that one which proves an improvement versus the null model [36]. The null model is considered a good reference because it includes no independent variables. In this study, different tests were used to evaluate LR models, LRT, and score test. These tests compared the difference between the −2LL estimate for the given model and the −2LL estimate for the null model. This difference is the Chi-square statistic with the same df for the two tests. The model with the lowest −2LL is considered the best for the fitted dataset. Fortunately, the two tests usually have the same conclusions and statistically approved by many authors [14,37-39]. The results of LRT, score test and the values of −2LL were shown in Table-6. The Chi-square test statistics represented the differences between two −2LL for LRT. The LRT and score test results were highly significant (p<0.01) for Model 3 (univariable logit model of species) and Model 5 (multivariable LR model). This implies that the addition of species into Model 3 resulted in an increase of Chi-square values (χ^2^=22.429, df=1) for LRT and χ^2^=121.648, df=1 for score test to be highly significant (p<0.01), indicating that a model with species was more effective than the null model. Similarly, the multivariable LR model was highly effective than null model, with the greatest increase in χ^2^ values (χ^2^=26.330, df=7) for LRT and χ^2^=25.449, df=7 for score test. The LRT and score test results for other models were non-significant (p>0.05), suggesting that these models (Model 1, 2, and 4) were not improved over the intercept-only model, and their predictors had no influence on the BVDV seroprevalence.

**Table-6:** Overall models evaluation and goodness of fit statistics.

Model	Test	χ^2^	df	p value	-2Log likelihood
Null model					945.442
Model 1 (gender only)	Overall model evaluation				944.963
	Likelihood ratio test	0.479*	1	0.489	
	Score test	0.480	1	0.488	
	Goodness of fit test				
	Hosmer and Lemeshow test	-	-	-	
Model 2 (age only)	Overall model evaluation				945.203
	Likelihood ratio test	0.240	2	0.887	
	Score test	0.239	2	0.887	
	Goodness of fit test				
	Hosmer and Lemeshow test	0.005	1	0.998	
Model 3 (species only)	Overall model evaluation				923.013
	Likelihood ratio test	22.429	1	0.001	
	Score test	21.648	1	0.001	
	Goodness of fit test				
	Hosmer and Lemeshow test	-	-	-	
Model 4 (area only)	Overall model evaluation				942.411
	Likelihood ratio test	3.032	3	0.387	
	Score test	3.047	3	0.385	
	Goodness of fit test				
	Hosmer and Lemeshow test	0.002	2	1.00	
Model 5 (all predictors)	Overall model evaluation				919.113
	Likelihood ratio test	26.330	7	0.001	
	Score test	25.449	7	0.001	
	Goodness of fit test				
	Hosmer and Lemeshow test	6.740	8	0.565	

Another approach to assess the studied models was the value of −2LL estimated by the model. It was concluded that multivariable logit model (Model 5), which had, not only the smallest −2LL value (919.113), but also the highest drop of −2LL from 945.442 to 919.113, was the best for predicting BVDV outcome, compared to other models. The Hosmer and Lemeshow statistics for goodness of fit of LR models suggest a good fit of data by all models (p>0.05). However, the Hosmer and Lemeshow Chi-square values not calculated for Model 1 and Model 3 (Table-7), because of data restriction, small number of groups (2) in contingency tables, and subsequently zero degrees of freedom.

**Table-7:** A classification table and predictive accuracy of the full model (the cutoff point is 0.5).

Observed	Predicted		

	Disease		Percentage correct

	Positive	Negative	
Disease positive (events)	110	141	43.8
Negative (nonevents)	141	345	71.0
Overall percentage			61.7

Sensitivity=110/(110+141)=43.8%; Specificity=345/(141+345)=71.0%; False positive=141/(141+345)=29.01%; False negative=141/(110+141)=56.18%

#### Multivariable versus univariable LR models

To assess the causative effects and relationships between more than one predictor and the outcome, a construction of multiple LR models should be carried out, controlling for other independent variables [40-42]. The multivariable LR model was statistically evaluated, not only in relation to the null model but also with the other models. Table-8 shows a comparison of the multivariable LR model with other logit models. The results revealed highly significant differences (p<0.01) between the multivariable LR model with the three single-predictor models, including sex, age, and localities, suggesting that the addition of more predictors lead to a significant improvement. The only non-significant difference in −2LL was observed between the multiple predictor’s models and a single-predictor model with species, indicating that addition of independent variables other than species insignificantly (p>0.05) improved the predictive ability of the model. In conclusion, the current multivariable LR model would be suitable for predicting the BVD prevalence than did other models.

**Table-8:** A Comparison between the multivariable logistic regression with all univariable logistic models using the differences in the-2LL.

Model comparisons	Chi-square	df	p value
Multivariable versus gender only models	25.85	6	0.00024**
Multivariable versus age only models	26.09	5	0.00009**
Multivariable versus species only models	3.90	6	0.69021^NS^
Multivariable versus area only models	23.298	4	0.00011**

** Highly significant at 0.01 level of significance (p<0.01). ^NS^Non-significant at 0.05 level of significance (p>0.05). -2LL=-2 Log likelihood

#### Predictive accuracy of the multivariable LR model

Table-7 presents the output of classification table for the multivariable LR, which would be recommended as the best for the present dataset of BVD. At cutoff point 0.5, the overall percentage of correct classification was recorded to be 61.7%, which imply an improvement made by the model. The sensitivity %, specificity %, false positive %, and false negative % were 43.8%, 71%, 29%, and 56.18%, respectively.

## Conclusions

The results of this study show that BVD is prevalent in the cattle and buffaloes population of Egypt. Animal species appears to be a significant risk factor for BVD infections, while the other risk factors, i.e., age, sex, and, herd location had no significant impact on BVD seroprevalence. In addition, we present different statistical methods that were highly compatible to use, therefore, this study indicates that multivariable LR is recommended as an alternative to Chi-square test for both association and predictive statistics for BVD seroprevalence, and may aid in studying the epidemiology of BVD virus particularly if there is no previous history of infections in closely located farms or regions.

### Authors’ Contributions

All authors participated equally in the study plan and design. AMS, MME, and EE collected the samples from the farms and assisted in the laboratory work. SAM carried out the statistical analysis of data and reporting the results. All authors collaborated in writing, revising, and improvement of the article for publication.
